# Application of ZnO Nanoparticles in Sn99Ag0.3Cu0.7-Based Composite Solder Alloys

**DOI:** 10.3390/nano11061545

**Published:** 2021-06-11

**Authors:** Agata Skwarek, Olivér Krammer, Tamás Hurtony, Przemysław Ptak, Krzysztof Górecki, Sebastian Wroński, Dániel Straubinger, Krzysztof Witek, Balázs Illés

**Affiliations:** 1 Department of Marine Electronics, Gdynia Maritime University, 81-225 Gdynia, Poland; agata.skwarek@imif.lukasiewicz.gov.pl (A.S.); p.ptak@we.umg.edu.pl (P.P.); k.gorecki@we.am.gdynia.pl (K.G.); 2 Department of Microelectronics, Łukasiewicz Research Network—Institute of Microelectronics and Photonics, 30-701 Kraków, Poland; krysztof.witek@imif.lukasiewicz.gov.pl; 3 Department of Electronics Technology, Faculty of Electrical Engineering and Informatics, Budapest University of Technology and Economics, 1111 Budapest, Hungary; krammer@ett.bme.hu (O.K.); hurtony@ett.bme.hu (T.H.); daniel.straubinger@ett.bme.hu (D.S.); 4 Department of Condensed Matter Physics, AGH University of Science and Technology, 30-059 Kraków, Poland; wronski@fis.agh.edu.pl

**Keywords:** ZnO ceramics, nanoparticles, composite solder, grain refinement, reinforcement, wettability, thermal behavior

## Abstract

The properties of Sn99Ag0.3Cu0.7 (SACX0307) solder alloy reinforced with ZnO nanoparticles were investigated. The primary ZnO particle sizes were 50, 100, and 200 nm. They were added to a solder paste at a ratio of 1.0 wt %. The wettability, the void formation, the mechanical strength, and the thermoelectric parameters of the composite solder alloys/joints were investigated. Furthermore, microstructural evaluations were performed using scanning electron and ion microscopy. ZnO nanoparticles decreased the composite solder alloys’ wettability, which yielded increased void formation. Nonetheless, the shear strength and the thermoelectric parameters of the composite solder alloy were the same as those of the SACX0307 reference. This could be explained by the refinement effects of ZnO ceramics both on the Sn grains and on the Ag_3_Sn and Cu_6_Sn_5_ intermetallic grains. This could compensate for the adverse impact of lower wettability. After improving the wettability, using more active fluxes, ZnO composite solder alloys are promising for high-power applications.

## 1. Introduction

The transition to lead-free soldering technology in 2006 resulted in the widespread use of tin–silver–copper (SAC) solder alloys in microelectronics packaging, mostly Sn96.5Ag3Cu0.5 (SAC305) and Sn95.5Ag4Cu0.5 (SAC405) alloys [1]. They provided appropriate soldering properties. However, they have two significant drawbacks due to the relatively high silver content, namely their higher price and possible reliability problems. If the silver content is over 2.8 wt % Ag in the case of 0.5 wt % Cu, then an inappropriate rate of cooling (below around 1.5 K/s) during soldering can result in the formation of large, brittle, plate-like Ag3Sn intermetallic compounds (IMCs) in the solder joints, and they can also cause shrinkage defects [2]. Therefore, over the past 5–10 years, the aim has been to reduce the silver content in SAC solder alloys while keeping the melting point between 217 and 227 °C. A promising low-silver solder alloy is Sn99Ag0.3Cu0.7 (SAC0307), with the micro addition (<0.1 wt %) of additional metals (such as Bi, Sb, Ni, etc.) called SACX0307. These metals can improve wetting and mechanical behavior, which may have decreased due to the lower silver content [3].

The most novel solution for improving low-silver-content solder alloys is the use of ceramic reinforcement particles in the solder paste, which results in composite solder joints. The size of the reinforcement particles is usually in the submicron- or nanoscale. A wide range of ceramic particles has already been applied by others in the literature, such as TiO_2_, ZrO_2_, Al_2_O_3_, Fe_2_O_3_, Si_3_Ni_4_, SiC, La_2_O_3_, ZnO, etc. [4,5,6,7]. Usually, the reinforcements positively affect the quality and/or reliability of the solder joints. Because the mixed nanoparticles are not soluble in Sn, they may increase the melting temperature, which could cause various issues during soldering [8]. The reinforcement particles usually change the thermal and mechanical parameters of the solder alloys [9,10] via grain refinement and the modified grain boundary/interfacial characteristics [11,12]. Typically, the SAC alloy’s solder joint is composed of a β–Sn matrix and intermetallic compounds (IMCs), such as Cu_6_Sn_5_, Cu_3_Sn, and Ag_3_Sn [13]. IMCs are present both in the solder bulk as isolated islands and as an IMC layer (typically Cu_6_Sn_5_) between the Cu pad and solder alloy. The general effect of the reinforcement particles on the microstructural changes is related to the enrichment of ceramic particles at the grain boundaries [14,15], as they are not soluble in Sn. At the grain boundaries, ceramic particles can suppress the grain growth (both of β–Sn and any kind of IMCs). This results in a refined β–Sn matrix, finer IMCs in the solder joint, and a thinner IMC layer at the Sn–Cu interface [14,16]. Furthermore, the interphase spacing between Ag_3_Sn grains is also reduced, which is favorable due to the increase in the mechanical strength of the solder joints [16]. On the other hand, the reinforcement particles usually increase the solidus–liquidus point of the composite solder alloy, as they are not dissolved [15].

Reportedly, the most popular reinforcement material is TiO_2_. Shi et al. found that TiO_2_ pins the grain boundaries, blocking grain boundary sliding and decreasing dislocation mobility. These phenomena cause the hardening mechanism of the β–Sn matrix [17]. Tsao et al. showed that TiO_2_ nanopowders could significantly increase (by 25%) the tensile strength and improve the microhardness of SAC solder joints. However, the ductility of composite SAC solder joints can be decreased [14]. Ramli et al. [10] observed that an increase in the weight percentage of TiO_2_ ceramics in a composite solder alloy decreases the coefficient of thermal expansion (CTE). A lower CTE is advantageous for better thermal fatigue reliability of the solder joints [10,18].

Rajendran et al. [19] investigated the reliability of SAC305 solder alloy reinforced by ZrO_2_ nanoparticles with isothermal aging. They found that the nanoparticles not only suppress the IMC layer growth during the soldering process but also decrease the diffusion coefficient of Cu, which results in slower IMC layer growth during isothermal aging. Furthermore, the composite solder joints showed higher shear strength at the as-reflowed and aged state of the samples. Jie et al. [20] investigated the effect of α-Al_2_O_3_ nanoparticles on the solderability of SAC0307 solders. They found that α-Al_2_O_3_ improved the composite solder’s wettability because of the nanoparticle absorption at the surface of the Cu substrate, which decreased the surface tension. It was also reported as well that α-Al_2_O_3_ ceramic nanoparticles gathered at the surface of the IMC layer and blocked atomic migration in the solder joints during electrothermal loads; thus, the electrothermal reliability of the composite solder joints improved considerably [21]. Sharma et al. [22] studied the effect of La_2_O_3_ nanoparticles on the quality of SAC105 solder alloy. They found a significant improvement in the mechanical properties of the composite solder joints due to the refinement of the microstructure and the increased dislocation density by secondary phase strengthening. SiC and Si_3_Ni_4_ nanoparticles were also successfully used in different solder alloys. They increased the ultimate tensile and yield strength and improved the wettability [23,24].

ZnO could be a favorable reinforcement for low-Ag-content SAC alloys for fabricating composite solders. The most significant advantages of ZnO are its low price; good physical and chemical properties, such as its high melting point of 1975 °C; good thermal stability; and appropriate elastic modulus. In high-Ag-content alloys, such as SAC305 and SAC387, the melting temperature did not increase significantly (only with ~1–2K) with the addition of ZnO nanoparticles. Furthermore, the creep resistance, the yield stress, and the ultimate tensile strength of these composite solder alloys were improved [25,26,27,28]. Qu et al. [29] investigated the influence of ZnO nanoparticles on the wettability and interface morphology of SAC305. They found that the effect on the wettability and the reduction in the thickness of the IMC layer highly depends on the mass fraction of ZnO in the paste. Peng et al. [30] observed a decrease in the development of the IMC layer during isothermal aging in the composite alloy SAC305-ZnO (1 wt %). They found an approximately 18% and 10% improvement in the microhardness and shear strength, respectively. Despite the previous positive effects, ZnO tends to agglomerate in the solder paste (especially at a micrometer particle size), which can cause low solderability. Furthermore, it can be aggregated by the flux applied during the soldering process [30], noting that this effect also occurred frequently in the case of other ceramic reinforcements. Consequently, the ZnO ceramic particles generally improved the quality and reliability of custom SAC solder alloys.

Our aim was to create composite solder alloys from ZnO particles and from the novel SACX0307 alloy and characterize their solderability and their physical parameters. Because most previous studies have investigated only composite solder bulks, our further aim was to describe the composite solder joints’ influence on the thermal properties of power LED assemblies.

## 2. Materials and Methods

### 2.1. Solder Joint Preparation

Nanocomposite solder alloys were produced from SACX0307 (Sn99Ag0.3Cu0.7, Alpha Industries, Chantilly, VA, USA) solder paste and 1.0 wt % of ZnO nanoparticles with different sizes using the ball milling process. The primary particle sizes were 50 (Sigma-Aldrich 677450-5G, St. Louis, MO, USA), 100 nm (Sigma-Aldrich 544906-10G-5G, Sigma-Aldrich, St. Louis, MO, USA) and 200 nm (Sigma-Aldrich 96479-100G, Sigma-Aldrich, St. Louis, MO, USA). The mixing process was carried out for 10 min at 300 rpm using a planetary ball mill Pulverisette 5 (Fritsch GmBh., Idar-Oberstein, Germany) to obtain a homogeneous distribution of the nanoparticles in the solder paste. The reference samples are subsequently referred to as SACX0307 and the nanocomposite samples as SACX0307-ZnO (50, 100, and 200 nm).

**Figure 1:**
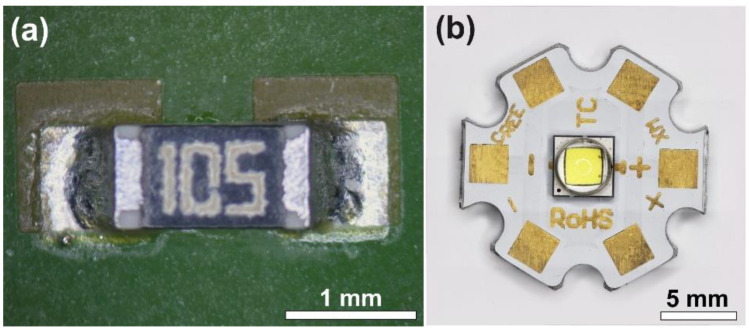
The samples tested: (**a**) soldered 0630 chip resistors; (**b**) the XMLBWT-02-0000-000HT20E7 power LED and the alumina substrate (Hirox optical microscope).

Wetting tests were performed on FR4 substrate with Ag surface finishing, and trial solder joints were fabricated with size 0603 (1.5 × 0.75 mm) chip resistors on the same substrate (Figure 1a). The performance of the composite solder alloys was also tested on the power LED application. For this purpose, alumina boards of 2.4 mm thickness were used as substrates (CREE Inc., Research Triangle Park, NC, USA). Contact surfaces were prepared from a 60 μm thick Cu layer with galvanic Ni/Au (2.5/0.25 µm) surface finishing. XMLBWT-02-0000-000HT20E7 power LEDs (CREE Inc., Research Triangle Park, NC, USA) were soldered onto the substrates (Figure 1b). Power LEDs were chosen as an application, as their thermal management is considerably influenced by the thermal and electric properties of the solder joints. In power LED applications, the thermal properties of the solder joint that ensures heat flow are essential.

The solder joints of both the resistors and the LEDs were prepared using classical surface mounting technology, reflow soldering. First, the solder paste was printed onto the substrates’ contact pads using stencil printing with a 125 µm thick stencil. The apertures of the stencil were reduced by 10% (compared to the contact pad size) to eliminate possible short circuit formation during the soldering process. The components were then placed onto the solder paste, and the solder paste was reflowed in an SMT 460C convection reflow oven (Essemtec AG, Aesch, Switzerland). A linear-type thermal profile was used: preheating (160–180 °C, 60 s), reflowing (213–247 °C, 40 s), and cooling (247–150 °C, 70 s). The soldering process took place in an air atmosphere.

### 2.2. Evaluation Methods

Initially, standard spreading tests were performed to investigate the wettability of the different composite solders. During the spreading test, round-shaped solder drops were deposited onto a continuous surface finish. After the reflow process, the largest wetted area was measured, and the surface quality of the solder spread was evaluated [31]. According to industry standards, the printed deposits were 5 mm in diameter, and the tests were repeated 16 times for each type of sample. The shear strength of the chip resistors was measured using a DAGE 2400 tester (Nordson DAGE ltd., Aylesbury, UK). Twenty resistors were tested at each solder type, and the means and deviations of the strengths were calculated.

For void identification, a two-dimensional (2D) static imaging technique and a three-dimensional (3D) MicroCT Imaging technique were used. All X-ray measurements were performed using GE Sensing & Inspection Technologies Phoenix X-ray Gmbh “Nanotom 180 N. ((GE Sensing & Inspection Technologies GmbH., Wunstorf, Germany)” The tomograms were registered on a Hamamatsu 2300 2300 pixel detector (HAMAMATSU PHOTONICS K.K., Shizuoka, Japan). The polychromatic beam was filtered using a 0.2 mm copper filter to reduce beam-hardening artifacts. The working parameters (for 2D and 3D examination) of the X-ray tube were I = 100 μA and V = 140 kV, and 1800 projections were taken. The exposure time was 500 ms and frame averaging of 10 (2D examination) and 5 (3D examination). The reconstructed images had a voxel size of 3.5 µm³. The measured objects were reconstructed with the aid of proprietary GE software datosX version 2.1.0 (GE Sensing & Inspection Technologies GmbH, Wunstorf, Germany)) using the Feldkamp algorithm for cone beam X-ray CT. The post-reconstruction was performed using VGStudio Max 2.1 (Volume Graphichs GmbH, Heidelberg, Germany).

Thermal and optical parameters of the LED assemblies were also measured: real thermal resistance (R_th_), electric thermal resistance (R_the_), and luminous efficiency (η_F_). A custom-designed LED test system was used to measure the diodes’ DC current–voltage characteristics, as described by Skwarek et al. [32]. Both the thermal and optical parameters of LEDs were measured simultaneously. The thermal parameter measurement method is an indirect electrical method. It means that the forward voltage of the LED is used as a thermosensitive parameter. Measurements were taken in two steps. In the first step, the LED was heated using a high forward current until a thermally steady state was reached. The electric power P_e_ (dissipated in the LED) was then measured. In the second step, the forward current was reduced to a low value, and the forward voltage was measured immediately after the current switched and at the steady state. The electrical thermal resistance R_the_ was calculated from the forward voltages at the beginning and at the end of the second measurement step and from the electric power. The real thermal resistance R_th_ was calculated taking into account the optical power P_opt_ and electric power P_e_ value [33,34]. During the measurements, the LED was situated in a light-tight chamber and mounted on the heat exchanger of a forced liquid cooling system, which guarantees the temperature of the substrate remains constant [35]. The optical efficiency η_F_ was calculated from the luminous flux and electric power ratio. Three samples of each type were analyzed.

The microstructure of the solder joints was analyzed on metallographic cross-sections. Two different scanning electron microscopes (SEMs) were used: an FEI thermal emission SEM (Thermo Fisher Scientific, Waltham, MA, USA) and a Thermo Scientific Scios 2 (Thermo Fisher Scientific, Waltham, MA, USA) ultra-high-resolution nonimmersion field-emission SEM. Backscattered electron (BSE) and secondary electron (SE) detectors were also used during the evaluations. The elemental composition of the samples was identified using energy-dispersive X-ray spectroscopy (EDX). A Thermo Scientific Scios 2 focused ion beam (FIB, Thermo Fisher Scientific, Waltham, MA, USA)) was used to prepare surface cuts on the cross-sections for detailed microstructural analysis.

## 3. Results and Discussion

### 3.1. Solderability and Thermal Behavior

**Figure 2:**
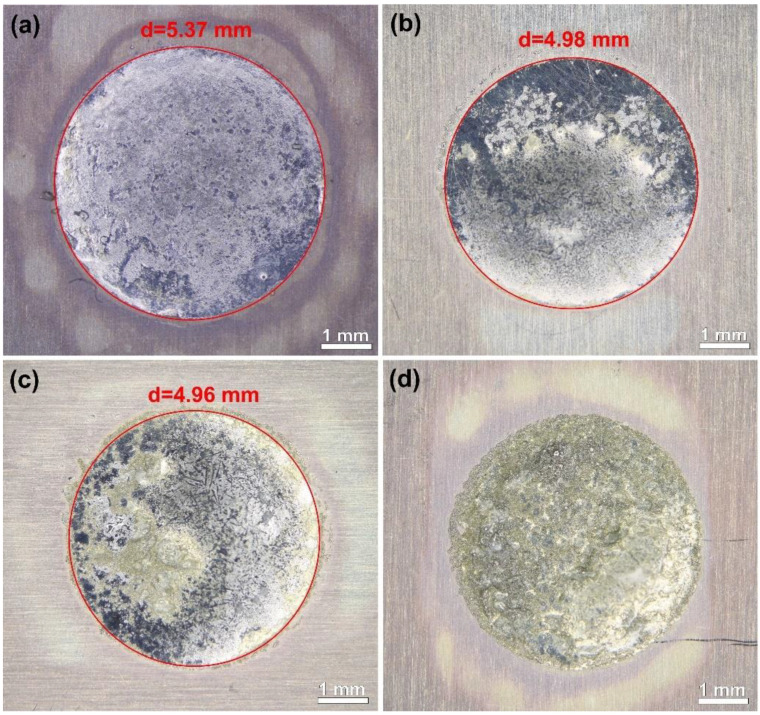
Results of the spreading tests: (**a**) SACX0307 reference; (**b**) SACX0307-ZnO-(200 nm); (**c**) SACX0307-ZnO-(100 nm); S (**d**) ACX0307-ZnO-(50 nm).

The wettability of solders is an important parameter for solder joint formation. It determines the effective area that the solder joint covers and affects the formation of intermetallic layers. Figure 2 shows examples of the results of the spreading tests in the case of the different solder alloys. The samples were cleaned with isopropyl alcohol to remove the flux residues before the evaluation. The cleaning process left light halos around the solder spots. The size of the reinforcement particles affected the wettability of the composite solder pastes. The wettability decreased with decreasing primary particle size. The SACX0307-ZnO-(200 nm) and (100 nm) pastes performed similarly but with a slightly lower wettability than the reference SACX0307. SACX0307-ZnO-(50 nm) showed even worse wettability, which is mainly observable on the rough surface of the solder spot (Figure 2d). The composite solder paste could not melt together to a continuous surface, so the wetting was not evaluable in the case of the nanoparticles with a primary particle size of 50 nm.

**Figure 3:**
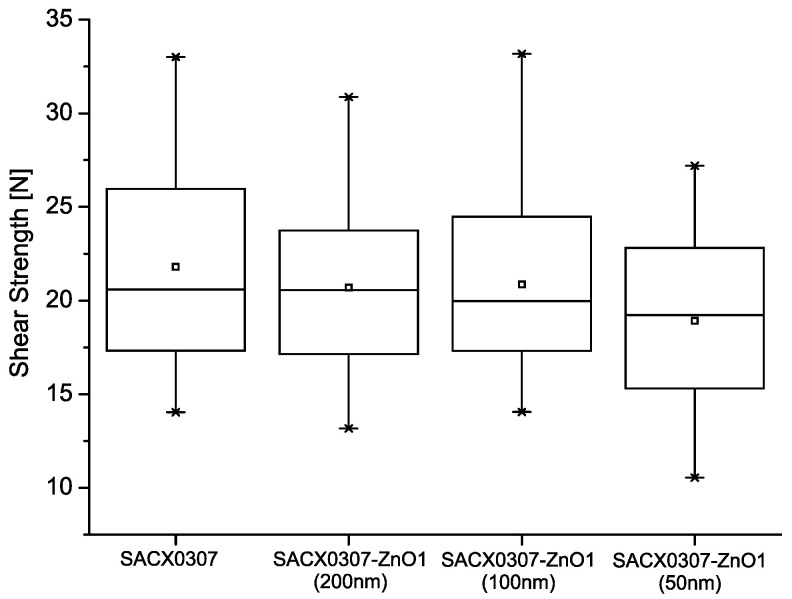
Shear strengths of the different solder joints.

The box plots of the shear strength values can be seen in Figure 3 The small squares represent the average, the horizontal lines indicate the median, the borders of the boxes indicate the ±σ standard deviation, and the crosses mark the min–max values. The wetting problems presented suggested a decrease in the shear strength of the solder joints, although the results disproved this assumption. The average shear strength of the SACX0307-ZnO-(200 nm) and the SACX0307-ZnO-(100 nm) (20.69 and 20.85N, respectively) solder joints almost reached the strength of the SACX0307 reference (21.88N). Additionally, SACX0307-ZnO-(50 nm) was only slightly weaker (18.92N). By statistical analysis, the strength of the different samples can be claimed identical at a significance level of 0.95.

**Figure 4:**
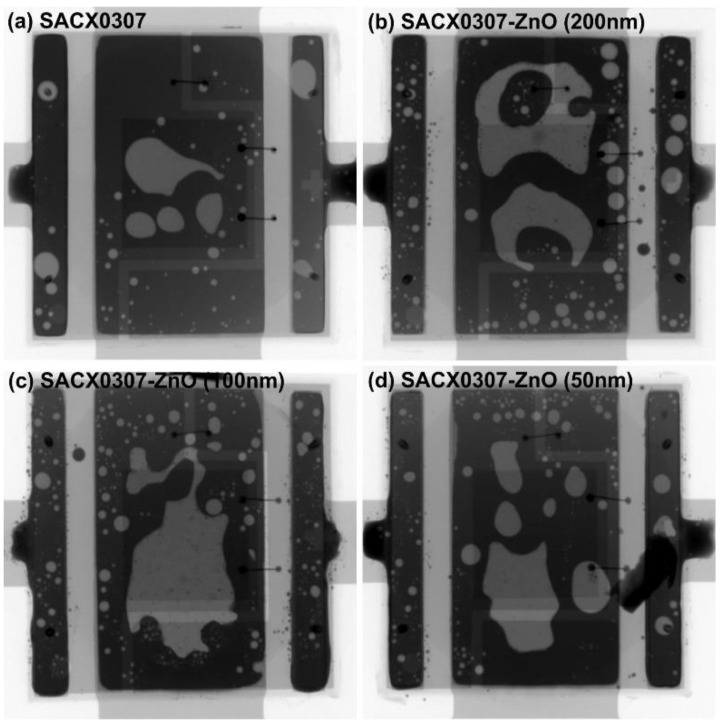
X-ray images of the different solder joints.

The soldering of large-sized power components is always challenging due to the increased void formation at the heat transfer pad, which stems from the fact that the flux’s outgassing is less efficient in such bounded solder joint structures. Therefore, the solder joints of the power LEDs were evaluated via X-ray as well. The void ratios of the solder joints were compared using a 2D X-ray system. The void ratio in the solder joints was determined by obtaining 2D X-ray images. The ZnO reinforcement particles always increased the void ratio in the solder joints, mainly at the heat transfer pad. The reference SACX0307 solder joints showed a void ratio of 11.7 ± 0.8%. The composite solder joints had a considerably higher void ratio: 25.2 ± 4% (200 nm), 26.3 ± 4% (100 nm), and 21.5 ± 3.5% (50 nm), respectively (Figure 4).

**Figure 5:**
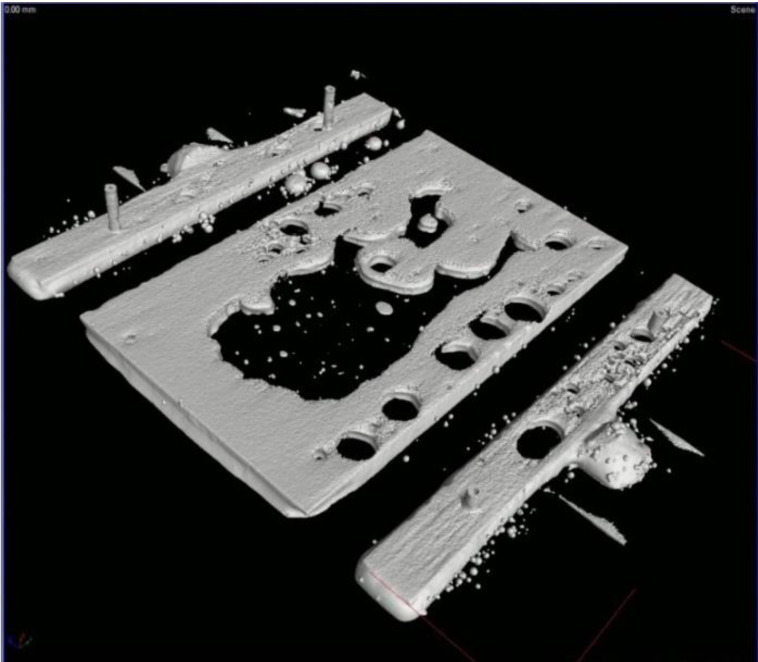
Three-dimensional (3D) CT image of a SACX0307-ZnO-(50 nm) solder joint.

Due to the increased void ratios of the composite solder joints, they were investigated using more accurate 3D CT measurements as well. Many solder balls were found inside the larger voids at the heat transfer pad in the composite solder joints. They are mainly visible on the 3D CT images (Figure 5) but also slightly visible in the 2D X-ray images (Figure 4b–d). The solder balls inside the voids suggest that the increased void formation was caused by the decreased wettability of the composite solder paste wettability. Peng et al. [30] observed solderability problems in micrometer-sized ZnO particles because they tended to agglomerate in the solder paste. According to our results, this problem can also occur in the case of the nanoparticle size range. ZnO is an amphoteric oxide, insoluble in water, but it will dissolve in most acids. One of the main components of solder paste is the flux that is based on acids. A reaction between ZnO and acids changes the surface tension of the paste, resulting in the worst wettability.

**Figure 6:**
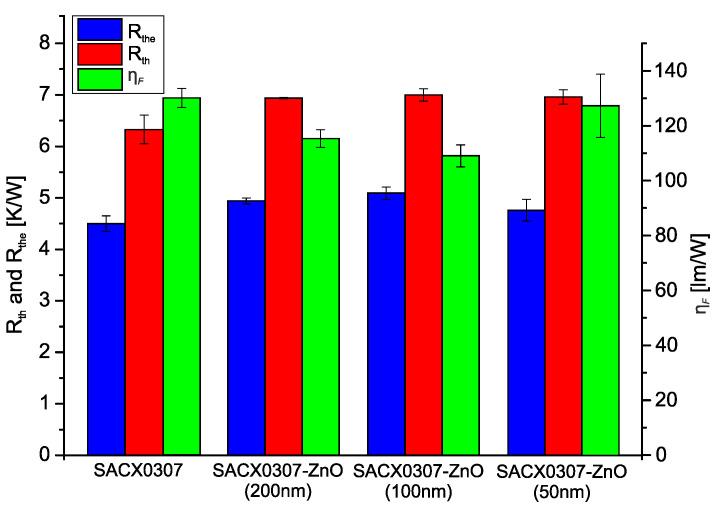
R_th_, R_the_, and η_F_ parameters of the different solder joints.

The increased voiding at the heat transfer pad suggested a decrease in the heat conduction ability of the nanocomposite solder joints, as the voids decrease the effective area of the solder joints where the heat is conducted. Therefore, the thermoelectric and optical parameters were determined. Figure 6 shows the real thermal resistance (R_th_), electric thermal resistance (R_the_), and luminous efficiency (η_F_) parameters of the different solder joints. The R_th_ and R_the_ parameters slightly increased, and η_F_ decreased in the ZnO nanocomposite solder alloys compared to the reference sample.

The increase in the heat resistances corresponds to the growth of the void in the different nanocomposite solder joints. However, the heat resistance increase ratio is much lower than the increase in the void ratios. The R_th_ and R_the_ parameters are not purely thermal types, as the excitation of the sample is electric. The microstructure of the solder joints affects the thermoelectric properties of the mounted components (LEDs), e.g., the IMC thickness itself has a high impact on thermoelectric parameters of the solder joints. Noh et al. proved that the increase in the IMC layer thickness causes an increase in the solder joints’ electric resistance [36]. Therefore, it is supposed that some positive microstructural changes in the nanocomposite solder joints could compensate for the negative effect of the increased void ratios.

### 3.2. Microstructural Evaluation

**Figure 7:**
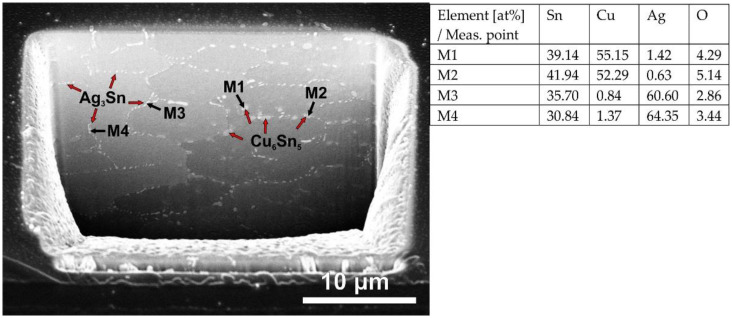
SEM-SE micrograph of an FIB cut on the SACX0307-ZnO-(200 nm) solder joint.

Microstructural evaluation of the solder joint includes the investigation of the area fraction of the IMCs in the solder bulk. Typical IMCs for Sn-rich solder alloys are Cu_6_Sn_5_, Cu_3_Sn, and Ag_3_Sn. Cu_6_Sn_5_ and Cu_3_Sn form a layer at the Sn–Cu interface (IMC layer) or precipitates in the solder matrix. The Ag_3_Sn forms only precipitations. Figure 7 shows the SEM-SE micrograph of a FIB cut on the SACX0307-ZnO (200 nm) solder joints at the solder bulk. After the FIB cutting, the sample was tilted 60° to be able to see the inside of the cut. It is visible that the Ag_3_Sn IMC particles grew at the grain boundaries of the Sn grains and formed a fine network in the Sn matrix. The Cu_6_Sn_5_ IMCs were located at the grain boundaries and occasionally inside the Sn grains as well. Therefore, the Ag_3_Sn IMC network can be used to detect the grain boundaries in the solder joints.

**Figure 8:**
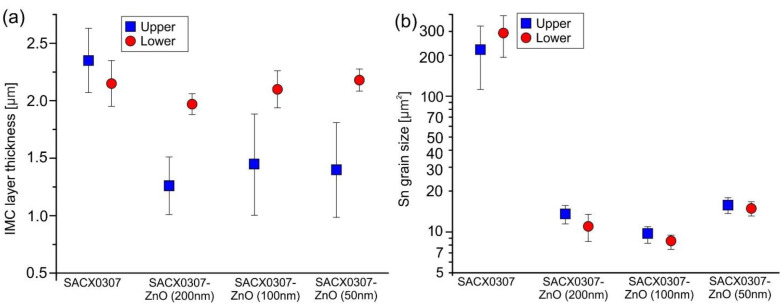
Microstructural Results 1: (**a**) average IMC thicknesses; (**b**) average Sn grain sizes.

Two regions of the solder joints were distinguished during the microstructural evaluation: the upper and lower parts. The upper part means the interface between the component (diode) and the solder joint. The lower part corresponds to the interface between the solder joint and the substrate. Both interfaces contain a Cu layer, which takes part in the formation of IMC layers. The IMC layer thicknesses and the Sn grain sizes are summarized in Figure 8. The averages were calculated from 10 cross-sections. The Sn grain sizes were evaluated according to their 2D projection size in the cross-section as surface areas in µm^2^.

**Figure 9:**
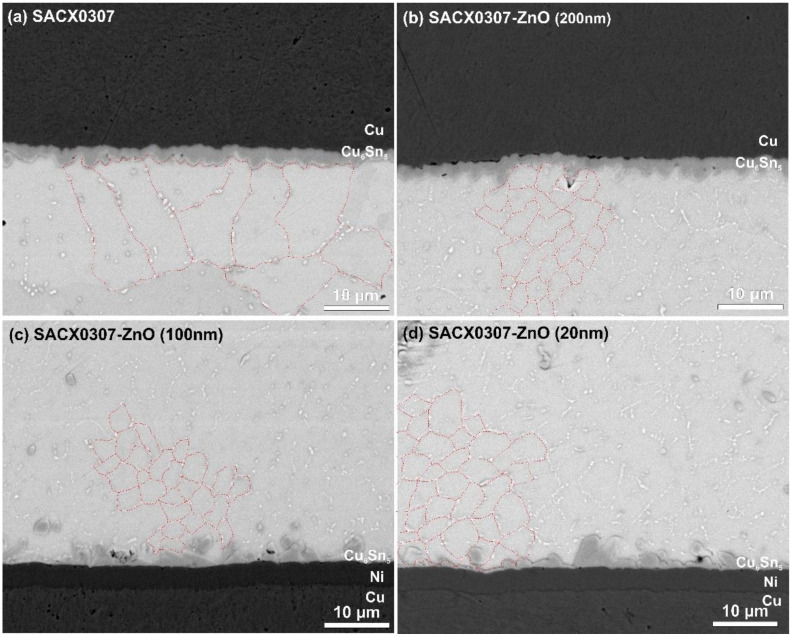
Sn grain and IMC layer structure of the different solder joints: (**a**) SACX0307; (**b**) SACX0307-ZnO-(200 nm); (**c**) SACX0307-ZnO-(100 nm); (**d**) SACX0307-ZnO-(50 nm).

The addition of ZnO ceramic into the SACX0307 solder alloy resulted in refinement of the Sn grains in each case, from ~200–300 µm^2^ to ~10–15 µm^2^. No significant differences were found between the composite solders, and the Sn grain refinement did not depend on the ZnO particle size in the range investigated (50–200 nm). The Sn grain boundaries are marked by red dashed lines according to the Ag_3_Sn network in the Sn matrix (Figure 9). The ZnO particles promoted a high nucleation density in the second phase in the eutectic colony during solidification.

This shows that ZnO nanoparticles have an increased ability to limit grain growth during solid-state cooling [27]. A possible explanation for the grain refinement is the surface adsorption theory. The ZnO particles—incorporated into solder—cannot wet the β–Sn matrix during the solidification process. They do not form IMCs in the solder matrix, and they are noncoarsening and nonreacting. The refinement of the microstructure is caused by high surface free energy on the solidified grain surfaces through the matrix, which adsorbs the ZnO nanoparticles during the solidification process [37]. No significant differences were found in Sn grain refinement between the upper and lower parts of the solder joint (Figure 8). This means that the distribution of the ZnO particles was most likely uniform in the solder joints.

In the IMC layers, the results differed significantly at the upper and lower regions of the solder joints. The addition of ZnO did not have any effect on the upper IMC layer growth. The average thicknesses were around 2 µm in all cases (Figure 9a,b). However, the growth of the lower IMC layer was significantly suppressed by the ZnO nanoparticles. They decreased the thickness to 1.1–1.4 µm. Some differences were found between the composite solder alloys. The largest (200 nm) ZnO nanoparticles provided the thinnest IMC layers. Because the distribution of the ZnO particles was uniform in the solder joints (according to the Sn grain refinement), the different growth of the IMC layer at the lower and upper part of the solder joints can be explained by other effects. The reason might be that the solder pads at the diodes did not contain the Ni barrier layer (opposite to the substrate). The Ni barrier layer decreases the Cu diffusion towards the solder joints. At the upper part of the solder joints, the ZnO ceramic could not have such a significant effect on the growth of the IMC layer than at the bottom part. At the upper part, there was no Ni barrier layer that could block the diffusion of Cu atoms from the solder pad.

**Figure 10:**
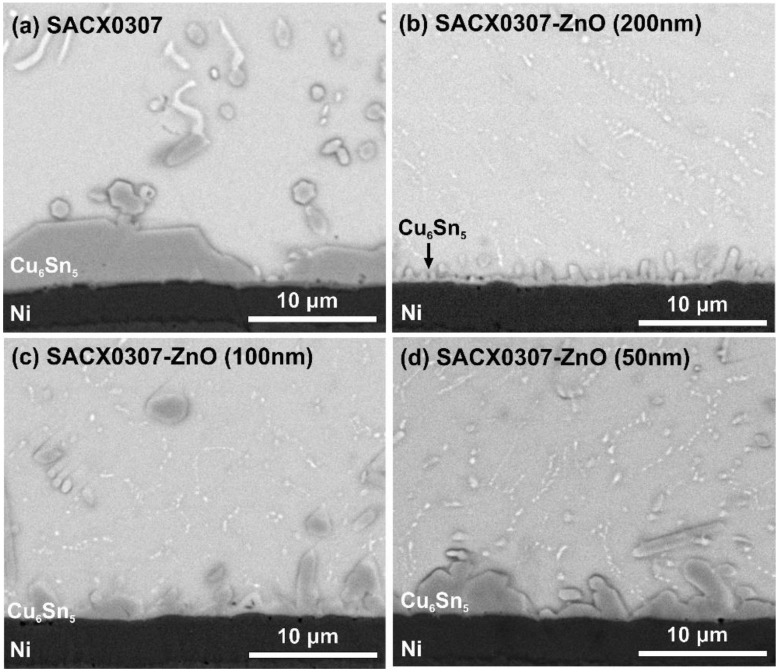
SEM-SE micrographs of lower IMC layer structures: (**a**) SACX0307; (**b**) SACX0307-ZnO-(200 nm); (**c**) SACX0307-ZnO-(100 nm); (**d**) SACX0307-ZnO-(50 nm).

The typical structure of the lower IMC layer in the different solder joints can be seen in Figure 10. The suppression effect of the ZnO (200 nm) particles on the growth of the IMC layer is the most visible (Figure 10a,b). The ZnO particles (with a size of 200 and 100 nm) not only decreased the average IMC layer thickness but also changed the morphology of the IMC layer from layer type to rough scallop type. In the case of the ZnO (50 nm), this effect was not as significant. The grain size of the Cu_6_Sn_5_ IMC layer is proportional to the evenness of the IMC layer [38], which has a considerable effect on the growth of the IMC layer. A thinner IMC layer contains more but smaller grains in a given volume than a thicker one, which results in a rougher IMC surface. During the growth of the IMC layer, the lattice diffusion of the Cu atoms is much more dominant through to the bulk of the grains than the grain boundary diffusion [38]. Therefore, the larger Cu_6_Sn_5_ grain size could result in a thicker IMC layer by the end of the formation of the solid–liquid-phase IMC. Consequently, the thinner lower IMC layer in the composite solder joints can be related to the fact that ZnO particles decrease the size of the Cu_6_Sn_5_ grain, which slowed down the growth of the IMC layer but resulted in a rougher IMC structure.

**Figure 11:**
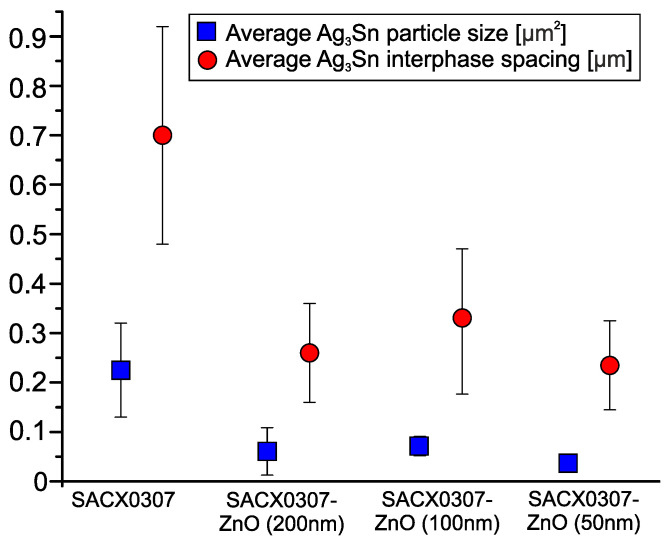
Average Ag_3_Sn particle size and interphase spacing.

As mentioned above, the other important IMC in this system is Ag_3_Sn. Figure 11 shows the average particle size of Ag_3_Sn and their average interphase spacing. The size of the Ag_3_Sn particle was also calculated according to their area fraction in the cross-sectioned plane, measured in µm^2^. As can be seen, the average size of the Ag_3_Sn particle and the spacing are significantly higher in the reference sample SACX0307.

The shear strength of the SAC solder joints is mainly determined by two parameters: the distribution of the Ag_3_Sn IMC particles in the solder matrix and the thickness of the IMC layer. Generally, a fine Ag_3_Sn mesh in the solder matrix and an IMC layer between 2 and 5 µm usually results in the best shear strength [39,40]. The presence of nanoparticles refined and dispersed the Ag_3_Sn IMC particles in the solder matrix and limited the growth of the Cu_6_Sn_5_ IMC layer much below 2 µm.

The ZnO nanoparticles and Ag_3_Sn IMC particles strengthen the solder matrix by generating dislocations that balance the expansion coefficient and elastic modulus differences between the solder matrix and the particles (ZnO and Ag_3_Sn) and block the dislocation movements [39,41]. Furthermore, the refined and dispersed Ag_3_Sn particles in the composite solders might cause strengthening during the load-transfer mechanism. The excellent interface bonding between the refined and dispersed Ag_3_Sn particles and the solder matrix results in a better load-transferring ability [39]. The yield stress of the solder joints (due to the piling of dislocations) during a load transfer depends on the interphase spacing between the refined and dispersed Ag_3_Sn particles [42]:

(1) $$\tau_{0}=\sqrt{\frac{G\cdot b\cdot \tau }{\pi \cdot k\cdot L}}$$

where *G* is the shear elastic modulus of the Cu pad, *b* is Burger’s vector, *k* is Poisson’s ratio, τ is the fracture stress of the Ag_3_Sn particles, and L is the average interphase spacing between the Ag_3_Sn particles.

According to (1), the smaller interphase spacing between the Ag_3_Sn particles yields better load-transfer ability, as it increases the τ. In the reference SACX0307 solder joints, the average spacing between Ag_3_Sn IMC particles was 0.7 ± 0.22 μm. The addition of 1 wt % ZnO nanoparticles decreased the interphase spacing between the Ag_3_Sn particles to 0.26 ± 0.1 μm, 0.33 ± 0.14 μm, and 0.24 ± 0.09 μm, respectively (Figure 11). Table 1 contains the calculated ratios of *L_SACX0307_/L_SACX0307-ZnO_* and their result in the *τ_SACX0307-ZnO_/τ_SACX0307_* ratios. The *τ* ratios can be used as a direct influential factor of the shear strength. According to the *τ* ratios, the composite solder joints were strengthened. However, the decrease in the thickness of the IMC layer (*h*) in the composite solder joints could cause the solder joints to weaken under certain circumstances.

It can be supposed that the dependence of the shear strength on the thickness of the IMC layer is nearly linear within a range of 1–2 µm [39,40]. Therefore, the ratios of the thicknesses of the IMC layer could be the other influential factor in the shear strength. According to the two significant factors mentioned, we can define a combined one (shear factor), which describes the opposite effect of the Ag_3_Sn refinement and the decrease in the thickness of the IMC.

(2) $$SF=\frac{\tau_{SACX0307-ZnO}}{\tau_{SACX0307}}\cdot \frac{h_{SACX0307-ZnO}}{h_{SACX0307}}$$

The IMC thickness (*h*) ratios and the shear factors (*SF*) for the different composite solders can also be seen in Table 1. If the average shear strengths (Figure 3) are compared to the shear factors (Table 1), a firm agreement can be found between them, which explains the effects of the ZnO nanoparticles observed. Some differences could be caused by the varying void content of the solder joints.

## 4. Conclusions

Different SACX0307-ZnO nanocomposite solder alloys were fabricated and characterized. The ZnO nanoparticles decreased the wettability, and this increased the voiding of the composite solder joints. The wetting decrease corresponded to the primary particle size of the nanoparticles (50–200 nm). The ZnO ceramic refined the Sn grains by one order of magnitude, refined the Ag_3_Sn IMC particles by ~70%, and decreased the thickness of the IMC layer by ~45%. These microstructural changes could compensate for the negative effect of excessive void formation, as the shear strength and the thermoelectric parameters of the composite solder joints remained almost the same, compared to the SACX0307 reference. The refining effects of the ZnO particles strengthened the load-transfer ability of the solder joints, but the decreased IMC thicknesses might equalize the shear strength of the solder joints near the reference level. With an improved wetting (using highly activated fluxes) of the ZnO composite solder alloys, their joints could exceed the quality of the reference SACX0307. Therefore, ZnO composite solder alloys are promising for high-power applications.

## Figures and Tables

**Table 1:** Influential factors on the shear strength.

Sample Type	L_SACX0307_ / L_SACX0307-ZnO_	τ_SACX0307-ZnO_ / τ_SACX0307_	h_SACX0307-ZnO_ / h_SACX0307_	SF [%]
ZnO (200 nm)	2.69	1.64	0.53	0.87
ZnO (100 nm)	2.12	1.45	0.72	1.04
ZnO (50 nm)	2.81	1.68	0.57	0.95

## Data Availability

The raw/processed data required to reproduce these findings cannot be shared at this time as the data also forms part of an ongoing study.
